# The role of comprehensive geriatric assessment in the identification of different nutritional status in geriatric patients: a real-world, cross-sectional study

**DOI:** 10.3389/fnut.2023.1166361

**Published:** 2024-01-08

**Authors:** Yanmin Ju, Xiaoye Lin, Kexin Zhang, Di Yang, Mengyu Cao, Huijing Jin, Jiyan Leng

**Affiliations:** Department of Cadre Ward, The First Hospital of Jilin University, Changchun, Jilin, China

**Keywords:** comprehensive geriatric assessment, nutritional status, Mini nutritional assessment, real-world, multivariable logistic regression analysis

## Abstract

**Background:**

Malnutrition is an often unrecognized problem, but it is common in older patients and leads to adverse outcomes.

**Aims:**

The purpose of this study is to analyze the prevalence of the risk of undernutrition in elderly patients and the correlation between CGA and nutritional status, and to determine the nutritional status of elderly patients.

**Methods:**

This is a real-world cross-sectional study of continuously enrolled elderly patients aged 65 years or older with a complete CGA database. CGA inventory was prepared by compiling and screening general information, body composition and blood biochemical results. MNA was also conducted for each elderly patient to screen for malnutrition. A multivariable logistic regression analysis was used to determine the association between the CGA and nutritional assessment.

**Result:**

The average age of the 211 selected elderly patients (160 men and 51 women) was 79.60 ± 9.24 years, and their ages ranged from 65 to 96 years. After controlling for confounders, patients with a history of PUD (OR = 2.353, *p* = 0.044), increased ADLs & IADLs scores (OR = 1.051, *p* = 0.042) or GDS scores (OR = 6.078, *p* < 0.001) may increase the incidence of the risk of undernutrition respectively, while an increase in BMI (OR = 0.858, *p* = 0.032) may lower the incidence of malnutrition risk. In addition, increased ADLs & IADLs scores (OR = 1.096, *p* = 0.002) or GDS scores (OR = 11.228, *p* < 0.001) may increase the incidence of undernutrition. However, increased MMSE (OR = 0.705, *p* < 0.001), BMI (OR = 0.762, *p* = 0.034), UAC (OR = 0.765, *p* = 0.048) and CC (OR = 0.721, *p =* 0.003) may decrease the incidence of undernutrition, respectively.

**Conclusion:**

The study found that the prevalence of risk of undernutrition in elderly patients was the highest. Risk of undernutrition was independently associated with peptic ulcer disease, ADLs & IADLs, GDS and BMI. However, we found that when the nutritional status reached the level of undernutrition, it was related to more factors, including ADLs & IADLs, MMSE, GDS, BMI, UAC and CC. Determining the level of malnutrition through CGA may help to prevent and intervene malnutrition as early as possible.

## 1 Introduction

Malnutrition is a chronic and often imperceptible process and identifying it at an early stage is crucial to reduce adverse outcomes (1). There are many related factors to malnutrition during the aging process, which may seriously affect the health status of the elderly (2). In addition, as people grow older, they are more likely to suffer from chronic diseases such as heart disease, cancer and diabetes, which will increase the risk of cognitive decline, weakness and disability. As is well known, improving nutrition will bring tangible benefits to the elderly, and many age-related diseases and conditions can be prevented, adjusted or improved by increasing nutritional levels (3). However, malnutrition in elderly patients has often been overlooked so far, so there is an urgent need to identify methods for malnutrition in order to prevent and treat it in a timely manner.

Comprehensive Geriatric Assessment (CGA) is a multi-dimensional and multidisciplinary diagnosis and treatment process, which assesses the physical diseases, psychological and functional abilities of the elderly; identifies high-risk patients; and further guides the disease prevention, treatment and follow-up (4, 5). It may also help to formulate long-term diagnosis and treatment plans (6). One study on health workers has shown that the CGA is related to nutritional indicators (7). In clinical practice, it has been found that elderly patients are willing to accept CGA, which may help identify the nutritional status of the elderly in clinical practice.

In this study, we hypothesized that CGA was related to nutritional status. If the hypothesis holds, we can use CGA to identify the different nutritional status of elderly patients, which can lead to early prevention and treatment.

## 2 Methods

### 2.1 Participants

This is a real-world cross-sectional study. We conducted an experimental study on consecutive outpatients and inpatients in the cadre ward department of the First Hospital of Jilin University from June 2020 to June 2021. The inclusion criteria were the elderly patients who had a complete CGA database and were 65 years old or older. The exclusion criteria were patients younger than 65 years old who did not participate in CGA or whose data were incomplete. The study was in line with the Helsinki Declaration and was approved by the Ethics Committee of the First Hospital of Jilin University (2021–478).

### 2.2 The process of CGA

The personnel conducting CGA receive specialized training in scale evaluation and instrument inspection (8). The detailed CGA assessment tools vary depending on institutions, medical environments and needs, but the definition of CGA is consistent, including the assessment of needs in multiple fields and the development of treatment plans (9). The measurement process of this study was personally tested by the same physician from the Cadre Ward Department of the First Hospital of Jilin University. Firstly, routine examinations should be conducted on enrolled patients. The collection of general information by face-to-face inquiry includes gender, age, educational background, smoking history, drinking history, sports and chronic diseases, such as cardiovascular disease (CVD), diabetes and peptic ulcer disease (PUD). The evaluation of the scale includes quality of daily life, cognitive test and geriatric depression scale. The evaluator dictates the survey content and records the patient’s answers. The next step for human body composition testing includes body mass index (BMI), upper arm circumference (UAC), calf circumference (CC), body fat percentage, and visceral fat area. Collect fasting venous blood from all patients the morning after admission and send them to the Laboratory Department of the First Hospital of Jilin University. After the results are reported, collect data on hemoglobin, albumin, prealbumin, triglycerides and low-density lipoprotein cholesterol (LDL-C). The experimental personnel collect and organize the data, and a total of 211 valid and complete data were obtained.

### 2.3 Detailed content of CGA

#### 2.3.1 General information

Collect personal information of patients, including gender, age, educational background, smoking history, drinking history, exercise (It refers to the patient walks for a total of more than 1 h per day and more than 5 days per week.) and chronic diseases (It refers to non-communicable chronic diseases, most commonly diabetes, obesity, metabolic syndrome, chronic kidney disease, cardiovascular disease, cancer and chronic respiratory diseases) (10). The patient has been diagnosed with the disease in the past and is in the process of disease monitoring and treatment.

#### 2.3.2 Quality of daily living

The living ability of elderly people was measured with the activities of daily living and instrumental activities of daily living (ADLs & IADLs). The ADLs & IADLs are important to older adults (11). ADLs includes physical care activities. These activities are essential for living in the social world. They enable people to obtain basic survival and well-being, such as eating, dressing, bathing and toileting; IADLs includes activities of daily life in families and communities, which usually are more complex than ADLs, such as making phone calls, taking medicine, financial management, purchasing groceries and housekeeping services (12). When the total score is greater than 16, it indicates that the patient has functional decline to varying degrees.

#### 2.3.3 Cognitive tests

Cognitive dysfunction was assessed using the Mini Mental State Examination (MMSE), a widely used cognitive test (13). The total MMSE score ranges from 0 to 30 (14), which consists of 30 items, including orientation tests, short-term memory and recall tests, attention and computing ability tests, naming tests, oral command tests, judgment tests and repeated pentagons tests (15). Cognitive impairment is evaluated based on differences in educational levels. A score of less than 18 was used to define cognitive impairment in participants without any formal education, less than 21 for those with six or fewer years of education, and less than 25 for those with more than six years of education (16, 17). All patients in this article have more than 6 years of education, the cut-off point for MMSE is 24, and a score of 24 or lower indicates cognitive impairment, which has been widely used as a screening tool in epidemiological research (17, 18).

#### 2.3.4 Geriatric depression

Depression was determined using the 30-item Geriatric Depression Scale (GDS) (19), which is a standardized self-reported questionnaire consisting of 30 dichotomous questions. Sum of the 30 items produced a score ranging from 0 to 30, with greater values indicating increased severity. A GDS score of 11 or above is considered depression (20, 21). One study on depression in elderly people in Amsterdam showed that the GDS-30 scale with a critical value of 11 is the most effective in screening for depression in terms of sensitivity and specificity for screening for severe and mild depression (22). And other studies have shown this cutoff has been shown to have 100% sensitivity and 84% specificity with other depression criteria (23).

#### 2.3.5 Body composition

Height and weight were measured according to clinical standards. BMI is calculated by dividing weight (kg) by height (m^2^).

Measure the UAC at the level of the thickest part of the biceps muscle when the upper arm drops naturally (accurate to 0.1 cm). Participants stood with their feet shoulder-width apart and measured the CC around the most prominent part of the gastrocnemius muscle (accurate to 0.1 cm). During the experiment, we tried our best to appease the patients and encourage them to cooperate with our measurements. We strictly followed the experimental steps and methods when measuring height, weight, UAC, and CC, proficiently used measuring instruments, accurately read, and conducted three measurements to minimize possible measurement errors and ensure the accuracy and reliability of the measurement results.

Body fat percentage and visceral fat area were measured by bioelectrical impedance analysis (BIA) using the Inbody S10 (Biospace, Seoul, Korea) (24).

#### 2.3.6 Blood biochemical results

Blood biochemical indicators such as hemoglobin, albumin, prealbumin, triglycerides, and LDL-C were obtained from the test results in electronic medical record.

### 2.4 Nutritional assessment

Nutritional status was assessed using the Mini Nutritional Assessment (MNA) questionnaire, which included 18 questions related to important factors of nutritional status, including food intake, weight loss, mobility, whether there is acute stress or disease, neurological problems, drug intake, BMI, UAC and CC. The highest score is 30 points. A score of 24 or more indicates a good nutritional status, a score between 23.5 and 17 indicates a risk of undernutrition, and a score below 17 indicates undernutrition (25). According to the MNA score results, the experiment was divided into three groups: normal nutrition group for scores ≥24, risk of undernutrition group for scores 17–23.5, and undernutrition group for scores <17.

### 2.5 Data analysis

The SPSS/WIN 23.0 software (IBM Corp., Armonk, NY, United States) and GraphPad Prism 8 (GraphPad Prism Software Inc., San Diego, CA, United States) were used for the statistical analysis. The Kolmogorov–Smirnov test was used to test the normality of the continuous variables. According to the distribution characteristics of data, continuous variables are expressed as mean ± standard deviation (SD) or median (interquartile range, IQR), and Student’s t-test or Mann–Whitney U-test was used to test the difference between the two groups. The categorical variables were expressed as absolute values and percentages, and chi-squared test were used to test the differences between the two groups. A multivariable logistic regression analysis was used to determine the correlation between PUD, ADLs & IADLs, MMSE, GDS, BMI, UAC, CC and MNA. Variables with *p* < 0.05 were selected for the multivariate analysis. The values of *p* < 0.05 were considered statistically significant.

## 3 Results

### 3.1 Baseline data of general characteristics, chronic diseases, ADLs & IADLs, MMSE and GDS

The average age of the 211 selected elderly patients (160 men and 51 women) was 79.60 ± 9.24 years, and their ages ranged from 65 to 96 years. As shown in Table 1, among the elderly patients, the incidence rate of undernutrition risk is the highest (43.60%), and older patients have poorer nutritional status. Among them, the median age of normal nutritional patients (*n* = 85, 40.28%) was 76.00, the median age of patients at risk of undernutrition (*n* = 92, 43.60%) was 81.50 and the median age of undernutritional patients (*n* = 34, 16.11%) was 90.00. Compared to patients with normal nutrition, the number of malnourished patients who exercise regularly is smaller. Moreover, as the nutritional status worsens, the ability of daily living of the elderly gradually declined, and the prevalence of related chronic diseases also gradually increased, especially CVD, PUD, depression and cognitive disorders (*p* < 0.05).

**Table 1:** Baseline data of general characteristics, chronic diseases, ADLs & IADLs, MMSE and GDS.

Variable	Normal Nutrition (85, 40.28%)	Risk of Undernutrition (92, 43.60%)	*p*	Undernutrition (34, 16.11%)	*p*
Gender, male, *n* (%)	70 (82.4)	66 (71.7)	0.094	24 (70.6)	0.155
Age, median (IQR), years	76.00 (68.00, 86.50)	81.50 (73.25, 88.00)	0.030	90.00 (87.50, 92.00)	0.001
Education background, *n* (%)			0.186		0.212
College degree or above	71 (83.5)	83 (90.2)		25 (73.5)	
Less than a college degree	14 (16.5)	9 (9.8)		9 (26.5)	
Smoke, *n* (%)	31 (36.5)	39 (42.4)	0.421	13 (38.2)	0.857
Drink, *n* (%)	35 (41.2)	39 (42.4)	0.870	15 (44.1)	0.769
Exercise, *n* (%)	59 (69.4)	57 (62.0)	0.297	11 (32.4)	<0.001
Types of chronic diseases, *n* (%)			0.007		0.024
≥3	24 (28.2)	44 (47.8)		17 (50.0)	
<3	61 (71.8)	48 (52.2)		17 (50.0)	
CVD, *n* (%)	31 (36.5)	47 (51.1)	0.050	20 (58.8)	0.026
Diabetes, *n* (%)	26 (30.6)	28 (30.4)	0.982	13 (38.2)	0.422
PUD, *n* (%)	11 (12.9)	26 (28.3)	0.012	10 (29.4)	0.033
ADLs & IADLs, median (IQR)	16.00 (14.00, 19.50)	20.00 (14.00, 28.00)	0.002	27.25 (20.00, 43.00)	<0.001
MMSE, median (IQR)	27.00 (25.00, 29.00)	27.00 (24.00, 28.00)	0.056	23.00 (19.00, 25.25)	<0.001
GDS, median (IQR)	7 (8.2)	36 (39.1)	<0.001	20 (58.8)	<0.001

IQR, interquartile range; CVD, cardiovascular disease; PUD, peptic ulcer disease; ADLs & IADLs, Activities of daily living and instrumental activities of daily living; MMSE, Mini-mental State Examination; GDS, Geriatric Depression Scale.

### 3.2 Baseline data of the anthropometric parameters and the blood biochemical indicators

As shown in Table 2, with the gradual decrease of BMI, UAC and CC, the nutritional status gradually deteriorates. Compared with elderly patients with normal nutrition, patients at risk of undernutrition and undernutrition have lower hemoglobin, albumin, prealbumin and triglyceride (*p* < 0.05).

**Table 2:** Baseline data of the anthropometric parameters and the blood biochemical indicators.

Variable	Normal Nutrition (85, 40.28%)	Risk of Undernutrition (92, 43.60%)	*p*	Undernutrition (34, 16.11%)	*p*
BMI, median (IQR), kg/m^2^	24.80 (23.05, 26.65)	24.00 (22.50, 25.50)	0.006	22.60 (24.80, 27.78)	<0.001
UAC, median (IQR), cm	27.40 (26.10, 28.80)	26.35 (25.40, 27.95)	0.004	24.80 (22.40, 26.03)	<0.001
CC, mean ± SD, cm	34.08 ± 3.77	32.40 ± 3.05	0.001	29.47 ± 2.90	<0.001
Body fat percentage, mean ± SD, %	30.32 ± 7.24	29.69 ± 7.52	0.570	30.07 ± 8.39	0.872
Visceral fat area, median (IQR), cm^2^	80.60 (64.00, 108.50)	79.10 (61.75, 89.95)	0.288	72.30 (55.70, 106.33)	0.436
HGB, mean ± SD, g/L	138.25 ± 19.93	130.12 ± 19.92	0.007	125.47 ± 18.30	0.002
Alb, median (IQR), g/L	38.40 (36.65, 42.05)	35.95 (34.60, 37.88)	<0.001	35.75 (32.73, 37.23)	<0.001
Prealbumin, median (IQR), g/L	0.23 (0.19, 0.29)	0.21 (0.18, 0.26)	0.120	0.19 (0.14, 0.26)	0.006
TC, median (IQR), mmol/L	1.41 (0.99, 1.94)	1.16 (0.83, 1.61)	0.011	1.03 (0.58, 1.67)	0.011
LDL-C, mean ± SD, mmol/L	2.93 ± 0.91	2.87 ± 0.85	0.660	2.75 ± 1.03	0.345

BMI, body mass index; IQR, interquartile range; UAC, upper arm circumference; CC, calf circumference; HGB, hemoglobin; Alb, albumin; TC, triglycerides; LDL-C, low density lipoprotein cholesterol.

### 3.3 Multivariable logistic regression analysis for risk of undernutrition.

**Figure 1:**
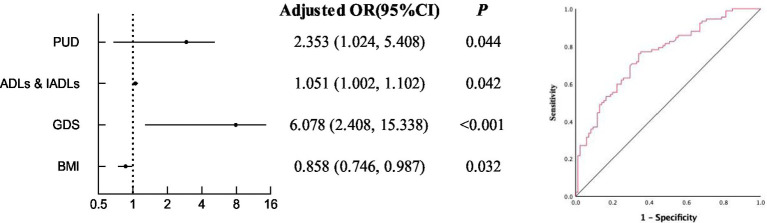
Multivariable logistic regression analysis for risk of undernutrition and the receiver operating characteristic of comprehensive PUD, ADL, GDS and BMI.

As shown in Table 3 and Figure 1, we analyzed the relationship between factors in CGA and risk of undernutrition. In model 1, after adjusting the age and the type of chronic diseases, patients with PUD were 2.353 times more likely to be at risk of undernutrition than those without PUD (95%CI: 1.024–5.408, *p* = 0.044). The probability of developing the risk of undernutrition increases by 5.1% for each 1- point increase in ADLs & IADLs (95%CI: 1.002–1.102, *p* = 0.042). In addition, depressed patients are 6.078 times more likely to suffer from the risk of undernutrition than those without depression (95%CI: 2.408–15.338, *p* < 0.001); In model 2, after adjusting for age, types of chronic diseases, PUD, ADLs & IADLs and GDS, for every 1 point increase in BMI, the elderly patients were 14.2% less likely to be at risk of undernutrition (95%CI: 0.746–0.987, *p* = 0.032). The area under the receiver operating characteristic of comprehensive PUD, ADL, GDS and BMI is 0.756.

**Table 3:** Multivariable logistic regression analysis for the risk of undernutrition.

	Crude		Adjusted	
Variates	OR (95%CI)	*p*	OR (95%CI)	*p*
Model 1				
PUD	2.650 (1.216, 5.776)	0.014	2.353 (1.024, 5.408)	0.044
ADLs & IADLs	1.063 (1.021, 1.107)	0.003	1.051 (1.002, 1.102)	0.042
GDS	7.163 (7.973, 17.259)	<0.001	6.078 (2.408, 15.338)	<0.001
Model 2				
BMI	0.842 (0.743, 0.955)	0.007	0.858 (0.746, 0.987)	0.032
UAC	0.835 (0.733, 0.952)	0.007	0.869 (0.752, 1.005)	0.059
CC	0.865 (0.790, 0.948)	0.002	0.944 (0.852, 1.047)	0.276
HGB	0.979 (0.964, 0.995)	0.009	0.985 (0.967, 1.004)	0.119
Alb	0.878 (0.813, 0.948)	0.001	0.940 (0.861, 1.026)	0.168
TC	0.669 (0.439, 1.022)	0.063	0.749 (0.465, 1.206)	0.234

PUD, peptic ulcer disease; ADLs & IADLs, Activities of daily living and instrumental activities of daily living; GDS, Geriatric Depression Scale; BMI, body mass index; UAC, upper arm circumference; CC, calf circumference; HGB, hemoglobin; Alb, albumin; TC, triglycerides; Model 1: Adjusted for age, types of chronic diseases; Model 2: Adjusted for age, types of chronic diseases, PUD, ADLs & IADLs, GDS.

### 3.4 Multivariable logistic regression analysis for undernutrition

**Figure 2:**
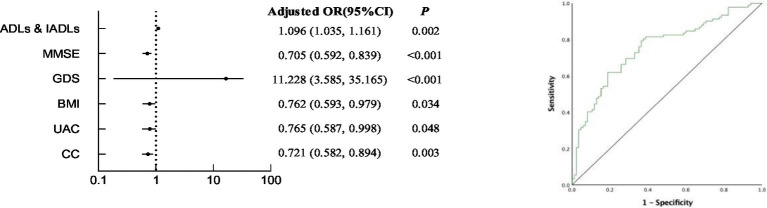
Multivariable logistic regression analysis for undernutrition and receiver operating characteristic of comprehensive ADLs & IADLs, MMSE, GDS, BMI, UAC and CC.

As shown in Table 4 and Figure 2, we analyzed the relationship between factors in CGA and undernutrition. In model 1, after adjusting the age, exercise and the type of chronic diseases, the probability of undernutrition increases by 9.6% for each 1-point increase in ADLs & IADLs (95%CI: 1.035–1.161, *p* = 0.002), the probability of undernutrition decreased by 29.5% for each 1 value increase in MMSE (95%CI: 0.592–0.839, *p* < 0.001). In addition, depressed patients are 11.228 times more likely to suffer from undernutrition than those without depression (95%CI: 3.585–35.165, *p* < 0.001); In model 2, after adjusting for age, exercise, types of chronic diseases, CVD, PUD, ADLs & IADLs, MMSE and GDS, for each 1-point increase in BMI, the probability of undernutrition decreased by 23.8% (95%CI: 0.593–0.979, *p* = 0.034), for each 1-point increase in UAC, the probability of undernutrition decreased by 23.5% (95%CI: 0.587–0.998, *p* = 0.048), or for each 1-point increase in CC, the probability of undernutrition decreased by 27.9% (95%CI: 0.582–0.894, *p* = 0.003). The area under the receiver operating characteristic of comprehensive ADLs & IADLs, MMSE, GDS, BMI, UAC and CC is 0.753.

**Table 4:** Multivariable logistic regression analysis for undernutrition.

	Crude		Adjusted	
Variates	OR (95%CI)	*P*	OR (95%CI)	*p*
Model 1				
CVD	2.488 (1.103, 5.613)	0.028	1.677 (0.684, 4.342)	0.287
PUD	2.803 (1.060, 7.411)	0.038	2.598 (0.807, 8.359)	0.109
ADLs & IADLs	1.128 (1.074, 1.185)	<0.001	1.096 (1.035, 1.161)	0.002
MMSE	0.690 (0.593, 0.804)	<0.001	0.705 (0.592, 0.839)	<0.001
GDS	15.918 (5.673, 44.666)	<0.001	11.228 (3.585, 35.165)	<0.001
Model 2				
BMI	0.721 (0.609, 0.853)	<0.001	0.762 (0.593, 0.979)	0.034
UAC	0.651 (0.533, 0.795)	<0.001	0.765 (0.587, 0.998)	0.048
CC	0.697 (0.601, 0.808)	<0.001	0.721 (0.582, 0.894)	0.003
HGB	0.968 (0.947, 0.989)	0.003	0.990 (0.960, 1.021)	0.536
Alb	0.799 (0.710, 0.898)	<0.001	0.931 (0.789, 1.099)	0.399
Prealbumin	0.000 (0.000, 0.049)	0.005	0.000 (0.000, 14.711)	0.141
TC	0.644 (0.360, 1.153)	0.139	0.962 (0.408, 2.269)	0.930

CVD, cardiovascular disease; PUD, peptic ulcer disease; ADLs & IADLs, Activities of daily living and instrumental activities of daily living; MMSE, Mini-mental State Examination; GDS, Geriatric Depression Scale; BMI, body mass index; UAC, upper arm circumference; CC, calf circumference; HGB, hemoglobin; Alb, albumin; TC, triglycerides; Model 1: Adjusted for age, exercise, types of chronic diseases; Model 2: Adjusted for age, exercise, types of chronic diseases, CVD, PUD, ADLs & IADLs, MMSE, GDS.

## 4 Discussion

Our study is the first to report the identification of different nutrition in elderly patients by CGA in the real world. Our results showed that many parameters of CGA are related to nutrition. In analyzing this relationship, we found that PUD, ADLs & IADLs, GDS, BMI, MMSE, UAC, CC were independently associated with nutritional status in elderly patients. The area under the ROC curve of the combined indicators under different nutritional states is greater than 0.7, therefore, the combination of corresponding indicators has certain diagnostic value for different nutritional states.

Scholars at home and abroad have been keen on the study of malnutrition and have found many factors related to malnutrition. Previous studies have demonstrated that PUD is associated with malnutrition (26). People with peptic ulcers such as duodenal ulcers often experience hunger or nocturnal abdominal pain, while people with gastric ulcers often experience postprandial abdominal pain, nausea, vomiting and weight loss (27).

Several large sample studies on the elderly have shown that depression is also related to malnutrition and these two diseases are common among the elderly in China (28, 29). And depression is a common mental illness characterized by decreased appetite, self-care, apathy and physical weakness. These characteristics may well explain the relationship between malnutrition and depression (30). In addition, the ability of daily living in elderly patients gradually decreases with age, and it has been reported that poor ability of daily living is associated with malnutrition (31). Others have found a link between nutritional status and cognition (32), the decline of cognitive function may affect the nutritional status, leading to a reduction in nutrient intake, and the resulting weight loss and malnutrition, which in turn may worsen cognitive function (33). Another important aspect is that there are many important indicators involved in nutritional assessment, such as BMI, UAC and CC (34, 35), which are valuable information for common nutrition-related diseases (36). Therefore, factors related to chronic diseases and body composition can affect the nutritional status of patients, and malnutrition can in turn affect the course of disease (37).

As age advances, the elderly are more likely to suffer from malnutrition or chronic energy deficiency, due to the deterioration of various physical functions, economic dependence and social isolation. Several small studies have demonstrated that the prevalence of malnutrition in the elderly ranges from 14 to 52% (38–40). It is well known that malnutrition is an underrecognized and undertreated problem throughout the health care system. Clinically, hospital malnutrition may lead to an increase in the number and severity of disease complications by increasing morbidity and mortality (41). The objective of this study was to analyze the factors of undernutrition and risk of undernutrition in order to identify different levels of nutrition. Through the identification of different nutritional conditions, early prevention and appropriate intervention can be carried out.

Previous studies have also analyzed the correlation between CGA parameters and nutrition. One of the cross-sectional studies of health professionals showed that CGA was associated with nutrition-related factors such as sarcopenia, sarcopenic dysphagia, cachexia. The appropriate care plan, including rehabilitation and nutrition management are provided according to the patient’s condition (7). Another study of older adults showed that age, drinking, chronic diseases, depression, BMI, ADLs & IADLs, number of recent falls, cognitive impairment, insomnia, low hemoglobin and albumin levels were independently associated with malnutrition. CGA can provide detailed information of elderly patients and is of great value for nutritional assessment (42). However, different from previous studies, this study was a real-world cross-sectional study that included elderly patients in outpatients and inpatients over a continuous period of time. In this study, we not only analyzed the general characteristics, scales and blood biochemical indicators of patients, but also included body composition factors such as UAC, CC, body fat percentage and visceral fat area. More importantly, this study specifically analyzed the different factors in CGA related to different levels of nutrition, so as to more specifically identify the current level of malnutrition in addition to more targeted prevention and treatment. This is a real- world cross-sectional study aimed at understanding the real living conditions of elderly patients and examining the relevance of geriatric diseases to the field of geriatric assessment, contributing to the prevention of many diseases and reducing the growing disease burden of the elderly.

There are some limitations to consider in our study. Firstly, since the patients included in our study are all patients in the cadre ward of the First Hospital of Jilin University, and these patients are groups with relatively high educational level, socioeconomic status and healthcare treatment level, and there is no significant difference. Considering that the impact on the experiment is small, there is not too much elaboration on this part of mixed factors in the experiment. In the future experiment, multi center, large sample research is very valuable, and excluding the possible confounding factors mentioned above may have more generalizability for the experimental results. Secondly, because this experiment is a small sample cross-sectional study, the results can only show the correlation between various factors and nutrition, which limits the ability to infer the causal relationship between malnutrition and observed results. Therefore, large sample cohort study in the future is very worthwhile and valuable and is very meaningful for malnutrition identification. Thirdly, based on clinical experience and literature review, it has been learned that medication use in the elderly may have an impact on their nutritional status. However, in this experiment, we did not include multiple medication use in the elderly. It is necessary to enrich the CGA database in future studies, such as including factors related to multiple medication use. Our CGA only included a small number of factors. In the future, it is necessary to further enrich the CGA content to have a more comprehensive understanding of nutrition-related factors in elderly patients.

## 5 Conclusion

The study found that the prevalence of risk of undernutrition in elderly patients was the highest. Risk of undernutrition was independently associated with peptic ulcer disease, ADLs & IADLs, GDS and BMI. However, we found that when the nutritional status reached the level of undernutrition, it was related to more factors, including ADLs & IADLs, MMSE, GDS, BMI, UAC and CC. Determining the level of malnutrition through CGA may help to prevent and intervene malnutrition as early as possible. And we found that the combination of corresponding indicators has significant diagnostic value for different nutritional states. It is suggested that the elderly should take appropriate exercise according to their physical conditions, improve their quality of life, improve their mood, eat high protein, low salt and low-fat food and regularly go to community hospital for medical treatment.

## Data availability statement

The original contributions presented in the study are included in the article/supplementary material, further inquiries can be directed to the corresponding author.

## Ethics statement

The studies involving humans were approved by the Ethics Committee of the First Hospital of Jilin University. The studies were conducted in accordance with the local legislation and institutional requirements. The participants provided their written informed consent to participate in this study.

## Author contributions

YJ, JL, and XL drafted the manuscript. YJ and XL revised the manuscript. KZ, DY, and MC drew the figures. XL, DY, and HJ were responsible for the data acquisition. YMJ and JYL performed the data analysis. YJ, HJ, and MC performed the statistical analyses. YJ, KZ and JL conceived of and designed the study. All authors contributed to the article and approved the submitted version.
